# Signaling properties of the human chemokine receptors CXCR4 and CXCR7 by cellular electric impedance measurements

**DOI:** 10.1371/journal.pone.0185354

**Published:** 2017-09-25

**Authors:** Jordi Doijen, Tom Van Loy, Wouter De Haes, Bart Landuyt, Walter Luyten, Liliane Schoofs, Dominique Schols

**Affiliations:** 1 Laboratory of Functional genomics & proteomics, Zoological Institute, KU Leuven, Belgium; 2 Laboratory of Virology and Chemotherapy, Rega Institute for Medical Research, KU Leuven, Belgium; Montana State University Bozeman, UNITED STATES

## Abstract

The chemokine receptor 4 (CXCR4) and 7 (CXCR7) are G-protein-coupled receptors involved in various diseases including human cancer. As such, they have become important targets for therapeutic intervention. Cell-based receptor assays, able to detect agents that modulate receptor activity, are of key importance for drug discovery. We evaluated the potential of cellular electric impedance for this purpose. Dose-dependent and specific stimulation of CXCR4 was detected upon addition of its unique chemokine ligand CXCL12. The response magnitude correlated with the CXCR4 expression level. G_αi_ coupling and signaling contributed extensively to the impedance response, whereas G_αq_- and G_βγ_-related events had only minor effects on the impedance profile. CXCR7 signaling could not be detected using impedance measurements. However, increasing levels of CXCR7 expression significantly reduced the CXCR4-mediated impedance readout, suggesting a regulatory role for CXCR7 on CXCR4-mediated signaling. Taken together, cellular electric impedance spectroscopy can represent a valuable alternative pharmacological cell-based assay for the identification of molecules targeting CXCR4, but not for CXCR7 in the absence of CXCR4.

## Introduction

G-protein-coupled receptors (GPCRs) are a large family of cell surface proteins that transduce a diverse array of extracellular stimuli (*e*.*g*. peptide and protein hormones, biogenic amines, and lipids) into intracellular signals, and eventually, biological responses. GPCR activation is of key importance in many developmental and physiological processes, and as a consequence, aberrant GPCR signaling contributes to many human diseases [1]. This, in combination with the ability to modulate GPCRs pharmacologically, make GPCRs the most popular and validated drug target class in medicine [2]. The subfamily of chemokine receptors consists of about twenty GPCRs, most of which display canonical signaling via the activation of heterotrimeric G-proteins, although some of them, referred to as atypical chemokine receptors, are devoid of G-protein-dependent signaling. The CXC chemokine receptor 4 (CXCR4) can only be activated by a unique ligand, the CXC chemokine ligand 12 (CXCL12), also known as stromal cell derived factor 1 (SDF-1) [3]. The CXC chemokine receptor 7 [CXCR7, also known as atypical chemokine receptor 3 (ACKR3)] has more recently been identified as an alternative receptor for CXCL12 that, given its high affinity for CXCL12 (~10 times higher than the affinity of CXCL12 for CXCR4) [4], is believed to act as a scavenger protein controlling the extracellular bioavailability of CXCL12 [5]. CXCR7 also binds a second chemokine ligand, the CXC chemokine ligand CXCL11 or I-TAC (Interferon-inducible T-cell alpha chemokine) albeit with ~20 times lower affinity compared to CXCL12 [6].

Both CXCR4 and CXCR7 have sparked interest as candidate drug targets. Besides its long-established role as a major co-receptor for HIV-1 viral entry and infection in CD4^+^ T-lymphocytes [7], CXCR4 is also involved in tumor cell growth, survival and metastasis of multiple types of human cancer [8], as well as several other diseases (*e*.*g*. inflammatory diseases, WHIM syndrome) [9]. CXCR4 is overexpressed in more than twenty types of human cancer, and the CXCL12-CXCR4 interaction in the tumor microenvironment controls tumor cell survival and resistance to chemotherapy [10]. Elevated CXCR4 expression levels have been associated with cancer metastasis and worse prognosis, both in hematological disorders and solid tumor cancers [7,11,12]. CXCR7 expression is also upregulated in several human cancers as well as their associated tumor vasculature, and has been linked to tumor cell proliferation, survival and neovascularization [13,14].

Unlike CXCR4 activation, binding of CXCL12 or I-TAC to CXCR7 does not lead to canonical signaling via heterotrimeric G-proteins (*e*.*g*. cytosolic calcium mobilization), but does induce β-arrestin-mediated signaling [15]. Furthermore, CXCR4 and CXCR7 are capable of forming both homo- and heterodimers [16,17]. In the latter case, the presence of CXCR7 modifies the CXCR4 signaling properties, including its ability to mobilize intracellular calcium [17,18]. Moreover, upon interaction with CXCR4, the recruitment of β-arrestin by CXCR7 was shown to be enhanced [15]. The *in vivo* significance of CXCR4-CXCR7 heterodimerization, however, remains poorly understood.

CXCR4 and CXCR7 are promising candidates for pharmacological targeting, which necessitates the development of drug discovery assays. In the case of CXCR4, G-protein-dependent calcium mobilization assays and receptor binding studies are often used as screening tools to identify receptor antagonists [19,20], whereas in the case of CXCR7, β-arrestin recruitment assays are the primary choice given its atypical signaling properties. Here, we evaluate in detail the use of cellular electric impedance analysis, a label-free and non-invasive method [21,22], as an alternative way to analyze CXCR4 and CXCR7 signaling. We show that this method is useful and sensitive for the detection of CXCR4-related signaling, but not for the detection of signaling downstream of CXCR7. By applying small molecule inhibitors, we investigated the contribution of G-protein signaling (G_αi_, G_αq_ and G_βγ_) downstream of CXCR4. In addition, we analyzed the mutual influence of CXCR4 and CXCR7 on their respective signaling in co-expression studies and co-culture experiments. To our knowledge, this is the first report demonstrating that cellular electric impedance spectroscopy is an appropriate alternative tool for the pharmacological profiling of the human chemokine receptor CXCR4.

## Materials and methods

### Cell lines and reagents

Human glioblastoma U87 cells expressing CD4 (U87.CD4) or CD4 and CXCR4 (U87.CD4.CXCR4), were kindly provided by Dr. D. R. Littman (Skirball Institute of Biomolecular Medicine; NY, USA). U87.CD4.CXCR7 cells stably expressing human CXCR7 were constructed as described previously [23], from U87.CD4 cells transfected with a pTEJ-8 plasmid containing human CXCR7 cDNA in co-transfection with a pPur vector (Takara Bio, Noji-Higashi Kusatsu City, Shiga Japan). The human colon cancer cell line HT-29 was obtained from Sigma-Aldrich, Saint Louis, USA (Cat. 91072201). These cells were cultivated in McCoy’s 5a medium (Sigma-Aldrich), supplemented with 10% fetal bovine serum (FBS; Thermo Fisher Scientific, Waltham, USA) and antibiotics (1 mM streptomycin and 1 mM penicillin; both from Sigma-Aldrich). Surface expression of CXCR4 was confirmed by flow cytometry (see below) throughout all experiments. Glioblastoma cell lines were cultivated in Dulbecco’s modified Eagle medium (DMEM; Thermo Fisher Scientific) supplemented with 10% FBS, 10 mM HEPES buffer (Thermo Fisher Scientific), and antibiotics [200 μg/ml geneticin (Thermo Fisher Scientific; CD4 selection) and 1 μg/ml puromycin (Sigma-Aldrich; CXCR4 and CXCR7 selection)]. CD4, CXCR4 and CXCR7 surface expression were confirmed by flow cytometry (see below) throughout all experiments. All cell cultures were maintained at 37°C and 5% CO_2_, and subcultivated every two to three days before reaching confluence.

Recombinant human CXCL12 and recombinant human CXCL11 were obtained from PeproTech (Rocky Hill, USA). AMD3100 octahydrochloride hydrate, gallein monohydrate, LY294002 hydrochloride, cytochalasin B, U0126 monoethanolate, digitonin, PAR1-AP, and thrombin from human plasma were obtained from Sigma-Aldrich, whereas YM-254890 was obtained from Wako laboratory chemicals (Neuss, Germany). Pertussis toxin (PTX) was obtained from Tocris (Bristol, UK) and PD98059 from Medchem Express (Sollentuna, Sweden). Dulbecco’s Phosphate Buffered Saline (DPBS) and 0.25% trypsine-EDTA (1X) were obtained from Thermo Fisher Scientific. Formaldehyde solution (37%) was obtained from Merck (Darmstadt, Germany).

### Flow cytometry

Approximately 800,000 cells were seeded per well of a 6-well plate in 2 mL complete medium and grown overnight. The next day, cells were washed once with DPBS with 2% FBS and resuspended to obtain 400,000 cells per 100 μL of DPBS with 2% FBS. This cell suspension (100μl) was transferred to a 5 mL round-bottom tube (Falcon) after which staining antibody was added (see below). After gentle mixing, the suspension was left for 30 min at ambient temperature before washing the cells with 2 mL DPBS with 2% FBS. Finally, cells were resuspended in DPBS with 1% formaldehyde and analyzed using BD FACSCanto™ II (BD Biosciences, San Jose, USA). Data were further processed using the BD FACSDiva Software and FLOWJO™ version 10.

Antibody staining: CXCR4 expression on U87.CD4.CXCR4 and HT-29 cells was demonstrated by applying 5 μL of allophycocyanin-labeled (APC-labeled) mouse anti-human CD184 (i.e. anti-human CXCR4; clone 12G5, cat: 555976, BD Pharmingen™, AB_398616) and 5 μL APC-labeled mouse anti-human IgG_2a_ (clone: X39, κ isotype control, cat: 340473, BD Biosciences, AB_400517). CXCR7 expression was monitored on U87.CD4.CXCR7 cells using 2.5 μL phycoerythrin-labeled (PE-labeled) mouse anti-human CXCR7 (clone: 10D1-J16, cat: 391404, Biolegend^Ⓡ^, San Diego, USA, AB_2565681) and 5 μL PE-labeled mouse anti-human IgG_2a_ (clone G155-178, κ isotype control, cat: 555574, BD Biosciences, AB_395953). U87.CD4.CXCR4 cells transfected with increasing amounts of CXCR7 plasmid DNA were co-stained with 2.5 μL PE-labeled mouse anti-human CXCR7 and 5 μL APC-labeled mouse anti-human CD184. APC-labeled mouse anti-human IgG_2a_ (5μl) and PE-labeled mouse anti-human IgG_2a_ (5μl) were used as isotype control. Unstained control samples (100 μL cell suspension to which no antibody was added) were included in each experiment.

### Purification of CXCR4-positive cells

To purify CXCR4-positive cells, Dynabeads™ sheep anti-mouse IgG (Invitrogen, Carlsbad, USA), a magnetic holder, and purified mouse anti-human CD184 (clone 12G5, cat: 555972, BD Pharmingen™, AB_396265) were used. Approximately 10x10^6^ cells were centrifuged (5 min, 400 g), and washed once in DPBS supplemented with 2% FBS. Cells were then incubated for 30 minutes in 1 mL DPBS + 2% FBS, to which 25 μL of the purified antibody was added. Cells were washed twice in DPBS + 2% FBS (5 min, 400 g), and resuspended in 6 mL DPBS + 2% FBS. In the meantime, 10x10^6^ beads were washed twice in DPBS + 2%FBS according to the manufacturer’s instructions, and finally resuspended in 3 mL DPBS + 2% FBS. The cell and bead suspensions were gently mixed, and the mixture was incubated for 30 min with constant mixing on a rotator. The mixture was washed twice witch DPBS + 2% FBS (magnetic holder) and the beads with cells were resuspended in 10 mL selective McCoy’s 5a medium supplemented with 10% fetal bovine serum (FBS), 1 mM streptomycin, and 1 mM penicillin.

### Cell transfection

Human glioblastoma U87.CD4.CXCR4 cells were transfected with a pBABE-Puro plasmid encoding CXCR7 (pBABE-Puro CXCR7). The amount of plasmid DNA in each transfection was kept constant by adding appropriate amounts of “empty” plasmid (pBABE-Puro EV). Transfections were performed using the FuGENE^Ⓡ^HD Transfection Reagent (Promega, Madison, USA) following the manufacturer’s protocol [24]. The day prior to transfection, U87.CD4.CXCR4 cells were seeded at 500,000 cells/well in a 6-well plate in 2 ml complete growth medium. For each transfection, a total amount of 3 μg of plasmid DNA was applied. Different ratios of pBABE-Puro CXCR7 and pBABE-Puro EV were diluted in opti-MEM (Thermo Fisher Scientific). Nine μL of the transfection reagent was carefully added to this mixture (100 μl final volume). After 15 min incubation, the mixture was added in a dropwise manner to the cells. Two days after transfection, cells were trypsinized, seeded for further experiments in plates with embedded gold electrodes (see below), and grown overnight. Cells were always used 3 days post-transfection, and receptor expression was also checked by flow cytometry on that day.

### Cellular electric impedance spectroscopy

The xCELLigence Real Time Cell Analyzer (RTCA) DP device (ACEA biosciences, San Diego, USA) uses well plates with embedded gold electrodes to sense cell behavior. It measures the impedance of an alternating current that runs through the wells. The magnitude of the impedance depends on the electrode configuration, the ion concentration, and on whether there are cells present in the well or not. A higher cell density will increase the impedance’s magnitude. The impedance also depends on the cell status *i*.*e*. cell adhesion and cell morphology. It is reported that impedance measurements can detect 1nm changes in vertical cell motion and thus are very sensitive [21]. Since GPCR activation can alter cell adhesion and can induce cytoskeletal rearrangements such as changes in the actin cytoskeleton [25–27], it can be detected using cellular impedance. The impedance response is therefore the sum of the cellular events elicited upon receptor activation that result in detectable changes in the cell status. The output of the xCELLigence device is Cell Index (CI), a quantitative measure of the cell status given by the equation: $CI=\max_{i=1,\ldots ,N}\left(\frac{R_{cell}(f_{i})}{R_{b}(f_{i})}-1\right)$. The CI uses the resistance component (R) of the impedance in which R_cell_(f) and R_b_(f) represent the frequency-dependent electrode resistances with and without cells (blank measurement), respectively. The three frequency points N used for CI calculation during our experiments are 10, 25, and 50 kHz [28]. Thus, an increase in CI can for instance result from enhanced cell attachment while a decrease in CI can result from a decrease in cell adhesion. E-Plate VIEW 16 PET (ACEA biosciences, San Diego, USA) with embedded gold electrodes were coated with 100 μL 0.1% gelatin in DPBS for 2 hours at ambient temperature. After washing the wells with DPBS, 50 μL/well of complete growth medium was added, and a blank measurement was performed using the xCELLigence device. After the blank measurement, 2x10^4^ cells (U87) or 6x10^4^ cells (HT-29) were seeded in each well in a final volume of 100 μL per well. Plates were left at ambient temperature for 20 min before being placed in the xCELLigence device at 37°C. Overnight growth (18–20 hours incubation time) was monitored using the RTCA device until a steady state was observed. Twenty μL of medium was carefully removed from the wells without disturbing the cell monolayer, and cultures were allowed to restabilize for one hour. Using an automatic multichannel pipet, 20 μL of compound (5x concentrated, dissolved in serum-free medium) was added to the wells, after which the CI was monitored every 5 seconds during a 1 hour compound pre-incubation phase. The compounds included AMD3100 octahydrochloride hydrate, gallein monohydrate, LY294002 hydrochloride, cytochalasin B, U0126 monoethanolate, and PD98059. In case of testing YM-254890 and Pertussis toxin, these compounds were pre-incubated overnight and the CI was measured every 20 minutes. Next, 25 μL of ligand (CXCL12 or CXCL11, 5x concentrated, dissolved in serum-free medium) was added to each well and the CI was recorded every 5 seconds during an interval of 1–2 hours. To the (negative) control wells, only serum-free medium was added. Experimental files were exported as Excel files, and data were further processed using Matlab R2016b from Mathworks^Ⓡ^,Natick, USA.

Similar procedures were followed for the ECIS Z instrument (Applied Biophysics, NY, USA). Like the xCELLigence device, the ECIS Z station measures the resistance component of the electric impedance. However, ECIS Z does this within a broader frequency range (100 Hz– 256,000 Hz) and does not translate the resistance readout into a CI formula [29].

### Calcium mobilization assay

CXCL12-induced calcium fluxes were measured using the FLIPR Tetra (Molecular Devices, Sunnyvale, USA) as described previously [19] with only minor adaptations. HT-29 cells were seeded at 6x10^4^ cells per well instead of 2x10^4^ cells per well for U87.CD4.CXCR4 cells. In addition, no gelatin coating was used for HT-29. After overnight growth, cells were loaded with the calcium sensitive dye Fluo-2 AM for 45 minutes. U0126, LY294002, cytochalasin B, AMD3100, gallein, and PD98059 were incubated for 1 hour with the cells before addition of CXCL12. Compounds that had to pre-incubate overnight (PTX, YM-254890) were added manually to the cells the day prior to the experiment. After washing the cells as previously described [19], the cells were exposed to the compound for one hour. After compound pre-incubation, cells were stimulated with CXCL12. For each sample, the difference between the maximum and minimum percentage of baseline (*i*.*e*. mean relative light units in each well during a fixed time interval before compound or CXCL12 addition) was calculated with the ScreenWorks 4.0^TM^ software (Molecular Devices), and data were further processed using Matlab R2016b. Negative (*i*.*e*. untreated cells without CXCL12 stimulation) and positive (*i*.*e*. untreated cells with CXCL12 stimulation) control samples were included in each experiment.

### Statistical data analysis

Data were analyzed using Matlab R2016b. Normalization of the cellular electric impedance responses was always done to the point of ligand addition *i*.*e*. for each response, all CI values were divided by the value at the point of ligand addition. Besides normalization, the baseline response (without ligand) was subtracted from the ligand-induced responses. These two manipulations resulted in a baseline-corrected normalized Cell index. For all experiments, technical replicates were included. These were performed on the same day with the same cells and the same ligand preparation. Independent experiments (biological replicates) were performed on different days, on cells with a different passage number, using different CXCL12 aliquots. Statistical analysis was always performed using averaged data points from independent experiments.

#### Electric impedance responses of ligand dilution series

After normalization and baseline-correction, the maximum response (MAX) and the area under each curve (AUC) within the first sixty minutes after ligand addition were calculated for each ligand concentration. Within each experiment, at least two technical replicates were averaged and taken as one data point. Using non-linear least squares regression (nlinfit; Statistics Toolbox) in Matlab, with the Hill equation sigmoid as model function, the EC_50_ was calculated from the MAX and AUC in function of the ligand concentration. This was done for each of three independent experiments after which the mean EC_50_ value with its standard deviation was calculated and reported.

#### Electric impedance responses after pre-incubation with compounds

After normalization and baseline-correction, the MAX and AUC were determined for each response. Within each experiment, the values obtained from technical replicates (n≥2) for the ligand-induced response without compound pre-incubation (*i*.*e*. serum free medium added instead) were averaged. The resulting value was set to 100 as the reference value. MAX and AUC values of responses from wells pre-incubated with compound were expressed relative to 100. Three independent experiments were used to determine the mean and standard deviation. Within each experiment, at least two technical replicates were averaged. P-values were determined using one sample t-tests with null hypothesis stating that there is no difference between the calculated response values after compound pre-incubation and the reference response value 100. The Benjamini-Hochberg procedure was applied to correct for multiple testing with False Discovery Rate (FDR) = 0.1 using the p.adjust function in R studio 2016 (Boston, MA, USA). FDR = 0.1 means that less than 10% of the significant results were allowed to be false positive values.

#### Calcium mobilization responses with compound pre-incubation

Within each experiment, at least three technical replicates were run. The average response of the control runs, in which assay buffer was added instead of compound and instead of ligand, was subtracted from the other mean values. The averaged response of reference samples, in which assay buffer instead of compound was used for pre-incubation prior to ligand addition, was set to 100. All other mean values were set relative to this value. The mean and standard deviation were determined over three independent experiments (biological replicates). One sample t-tests and the correction for multiple testing were performed in a similar way as for the electric impedance responses with addition of compounds.

#### Electric impedance responses on co-expression cultures

In these cultures, CXCR4 and CXCR7 were co-expressed on the same cell. After normalization and baseline-correction (as described previously), the MAX and AUC were determined. The average CXCL12-induced response (n = 2) on U87.CD4.CXCR4 cells transfected with solely “empty” vector plasmid (no CXCR7 present) was set to 100% in each experiment and values obtained for cells transfected with increasing amounts of CXCR7 were calculated relative to this value. During each transfection experiment, three different quantities of CXCR7 plasmid DNA were transfected in U87.CD4.CXCR4 cells. In total, data from six independent transfection experiments were included in the regression analysis. The relative MAX and AUC values from each experiment were plotted against the corresponding percentage of CXCR7-positive cells, which was determined by flow cytometry as described previously. The relationship between the percentage of CXCR7-positive cells and the relative MAX and AUC was assessed using linear least square regression. The intercept was forced to 100 and an F-test was performed with the null hypothesis stating that increasing amounts of CXCR7 do not reduce the impedance response (y = 100).

#### Electric impedance responses on co-cultures

In these cultures, cells exclusively expressing CXCR4 or CXCR7 were co-cultured. Thus, the receptors were not co-expressed on the same cell. The response MAX and AUC from co-cultures with both U87.CD4.CXCR4 (X4^+^/X7^-^) and U87.CD4 (X4^-^/X7^-^) cells were taken as the reference and were set to 100 in each experiment. The response MAX and AUC from the corresponding co-culture with an equally large X4^+^/X7^-^ population but with an U87.CD4.CXCR7 population (X4^-^/X7^+^) instead of the X4^-^/X7^-^ population were set relative to the reference response. The total number of seeded cells remained constant. The mean and standard deviation were determined over three independent experiments and within each experiment at least two technical replicates were used. Linear least square regression was used to find the best fit with intercept forced to 100, and an F-test was performed in a similar way as described for the co-expression cultures. In the co-expression setting, the amount of CXCR7-positive cells equals the amount of cells positive for both CXCR4 and CXCR7, because CXCR4-positive cells were used for transfection. In the co-cultures, not all cells were CXCR4-positive. Therefore, the % of CXCR7-positive cells was determined by the ratio [X4^-^/X7^+^]/[X4^+^/X7^-^]*100.

#### Electric impedance responses on original and CXCR4-enriched HT-29 cultures

The values for the MAX and AUC were determined for all responses. The mean and standard deviation over two independent experiments were calculated. CXCR4 expression levels were similar in the two independent experiments as checked by flow cytometry. Within each experiment, four technical replicates were averaged. The calculated mean MAX and AUC value from the original HT-29 culture response were set to 100% and the MAX and AUC of the enriched HT-29 cells were set relative to this value. One sample t-tests were performed with null hypothesis stating that the response MAX and AUC of the enriched culture did not differ from the original culture’s response MAX and AUC which was set to 100.

#### Evaluation of pre-incubation phase

For each compound, the disturbance of the CI prior to chemokine addition was evaluated. Strong disturbance of the CI by a compound itself can influence the chemokine-induced impedance response and would hamper the interpretation of the chemokine-induced CXCR4-mediated impedance response afterwards. For this, the mean and standard deviation of the control response (i.e. only medium added during the pre-incubation phase) was determined. A trust interval was generated corresponding to three times the standard deviation calculated using four independent experiments, with four technical replicates measured in each experiment. In case the disturbance of the CI was bigger than three times the standard deviation of the mean control response subsequent to adding medium, the corresponding compound concentration was excluded from further analysis (**S5 Fig**).

## Results

### CXCL12 induces a specific and dose-dependent cellular electric impedance response on CXCR4-expressing cells while no such response is observed on CXCR7-expressing cells

**Fig 1:**
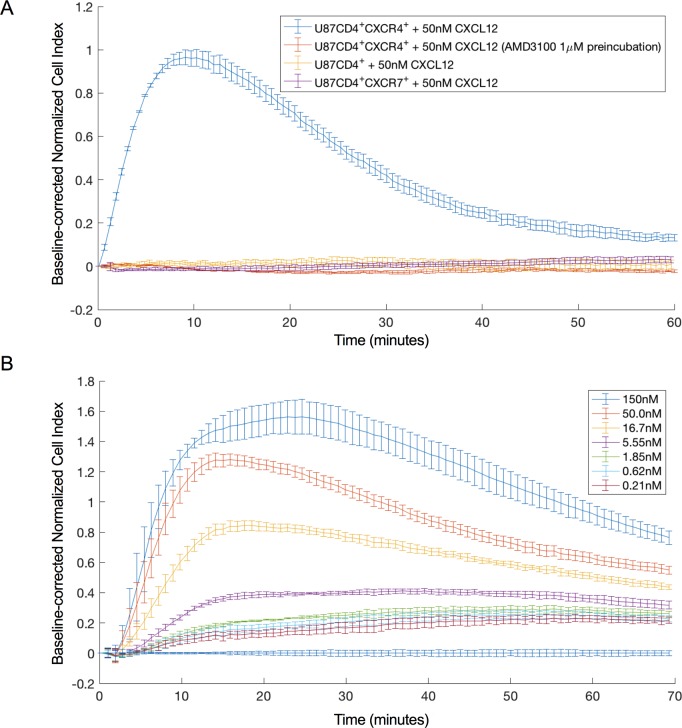
Impedance profile resulting from CXCL12-induced CXCR4 signaling. **(a)** CXCL12-induced impedance response on U87.CD4.CXCR4 cells with (orange) and without (blue) one hour pre-incubation with the specific CXCR4 antagonist AMD3100. CXCL12-induced impedance response on U87.CD4 (yellow) and U87.CD4.CXCR7 cells (purple). For each time point the mean CI value and standard deviation of 3 technical repeats within the same experiment are shown. **(b)** Dose-dependent response on U87.CD4.CXCR4 cells elicited by 0.2 nM—150 nM CXCL12. The figure depicts one out of three independent datasets used to calculate the mean EC_50_. The mean CI and standard deviation of 6 technical repeats are shown. Normalization and baseline-correction were done as described in the method section. For clarity, one in every 5 data points is plotted.

Using an xCELLigence device, the effect of CXCL12 stimulation on U87.CD4.CXCR4 and U87.CD4.CXCR7 cells, stably expressing CXCR4 and CXCR7, respectively, was investigated. Cells were seeded in gelatin-coated plates (E-Plate VIEW 16 PET), and incubated overnight while attachment of the cells to the well surface, and growth, were monitored (**S1 Fig**). A positive and transient CXCL12-induced CI response was observed for U87.CD4.CXCR4. The specificity of this response was demonstrated as it could be completely blocked by a one-hour pre-incubation with 1 μM (final concentration) of the CXCR4-specific antagonist AMD3100, and in addition, no response was observed on U87.CD4 cells lacking CXCR4 expression (**Fig 1A**). Applying a CXCL12 dilution series yielded a dose-dependent CI response on U87.CD4.CXCR4 cells, which permitted calculating the EC_50_ value for receptor activation based on the MAX and AUC within the time frame of sixty minutes immediately following CXCL12 addition. Both approaches yielded comparable EC_50_ values for receptor activation (32.4 +/- 7.4 nM and 39.4 +/- 0.6 nM, respectively) whereby the mean and standard deviation of the EC_50_ values were determined based on three independent experiments of which one of them is depicted in **Fig 1B**.

Unlike CXCR4, CXCR7 has previously been shown not to signal via heterotrimeric G-proteins, but it does recruit β-arrestin [30]. When the natural agonists for CXCR7 (i.e. CXCL12 or CXCL11) were applied on U87.CD4.CXCR7 cells, no response was detected with the xCELLigence device, even at concentrations up to 400 nM (**Fig 1A and S2 Fig**). We questioned whether the G-protein independent signaling events, if any, induced by CXCR7 activation could possibly be detected at other frequencies that are not within the detection range from the xCELLigence device. Therefore, additional experiments were performed on the ECIS Z device. The ECIS Z device also measures the resistance component of the impedance but can do this at frequencies ranging from 100 Hz to 256,000 Hz.

When the agonist-induced impedance response on U87.CD4.CXCR7 cells was recorded using an ECIS Z with 96W array station, even within this broad frequency range, no obvious CXCR7-related response was detected. Of interest, when the CXCR4 impedance response was analyzed at different frequencies on the ECIS device, it became clear that the maximum CXCR4 response was detected within a narrow frequency range that corresponds well with the default frequency settings (10, 25, and 50 kHz) of the xCELLigence DP device, used for CI calculation. In addition, the shape of the CXCR4 response profile did not change throughout the spectrum. All further experiments were conducted using the xCELLigence device for its convenience and fast data acquisition.

### CXCL12-induced response on endogenously expressed CXCR4

**Fig 2:**
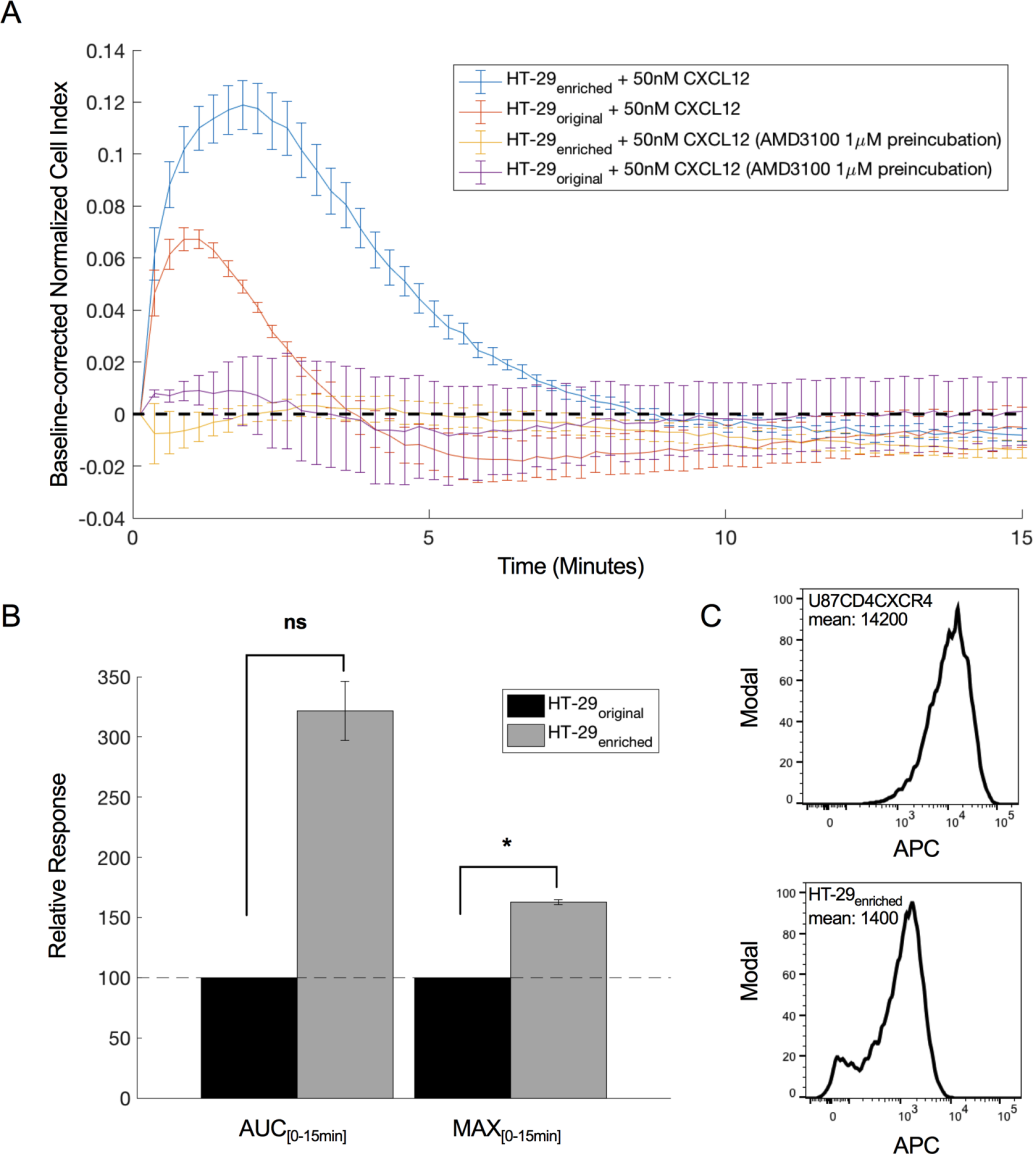
CXCL12-induced impedance response of endogenously expressed CXCR4 in HT-29. **(a)** Endogenous CXCL12-induced CI response on original (orange) and enriched (blue) HT-29 cells with 36% and 90% CXCR4 expressing cells, respectively. Responses from both cell populations can be blocked by a one-hour pre-incubation with 1 μM AMD3100 (yellow and purple graph). Mean and standard deviation are shown from four replicates within one experiment. For clarity, one in every 2 data points is shown. **(b)** Relative MAX and AUC of original (black) and enriched (grey) HT-29 cultures calculated using the response within the first 15 minutes after CXCL12 addition. Mean and standard deviation are calculated from two independent experiments. Within each experiment four technical replicates are averaged. P-values were determined using one sample t-tests with null hypothesis stating that there is no difference between the enriched response value and the original HT-29 response (100). * indicates significant differences from the original HT-29 response at significance level 0.05. The difference in AUC between original and enriched HT-29 response is not significant (ns; p = 0.07). **(c)** Comparison between mean APC-labeled anti-CXCR4 signal intensity of U87.CD4.CXCR4 cells and enriched HT-29 cells using flow cytometry.

CXCR4-mediated impedance responses were also studied in a cell line endogenously expressing the receptor. For this, expression of CXCR4 on HT-29 cells, a colon cancer cell line, was confirmed by flow cytometry, where ~ 36% of the cells appeared to be CXCR4-positive (**S3 Fig**), with little fluctuation over time. 50 nM CXCL12 evoked a small, but detectable impedance response on these HT-29 cells, that could be fully inhibited by AMD3100 (**Fig 2A**). When the population of CXCR4-positive HT-29 cells was enriched using Dynabeads™ (see methods), an enriched HT-29 population with ~90% CXCR4-positive cells was obtained (**S3 Fig**). An increased CXCL12-induced response, that could be blocked by one-hour pre-incubation with AMD3100, was detected on this enriched population (**Fig 2A and 2B**). Of note, neither for the original nor for the CXCR4-enriched HT-29 cells, was CXCL12 able to induce an eminent response in the calcium mobilization assay that was performed in parallel using a FLIPR Tetra system (**S6 Fig**).

### The CXCL12-induced cellular electric impedance response in CXCR4-expressing cells is dominated by PTX-sensitive G_αi_-signaling

**Fig 3:**
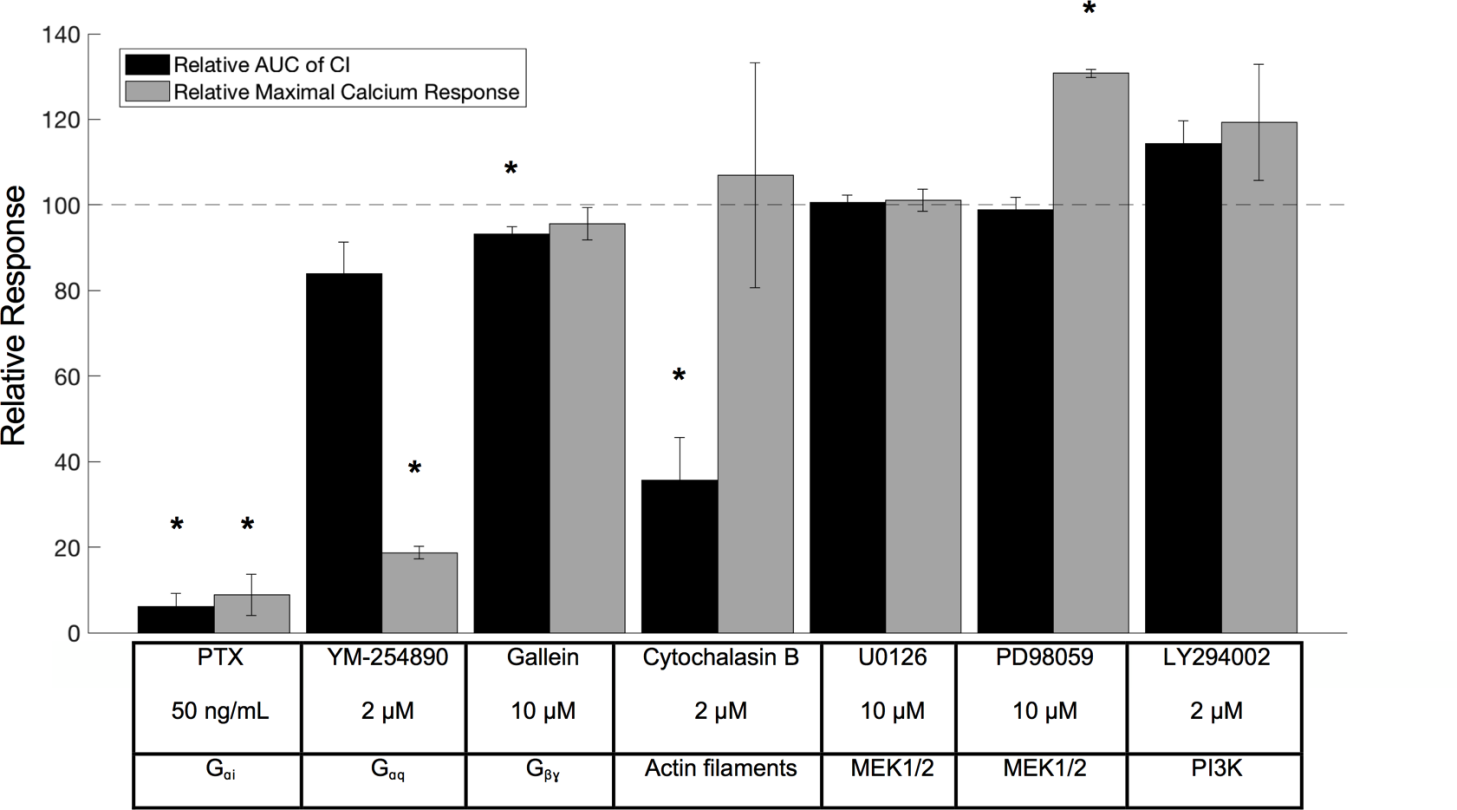
Effect of different compounds on the CXCL12-induced CXCR4 impedance response. Relative AUC within the first 60 minutes after ligand addition (50 nM CXCL12) (black) and calcium response intensity (grey) induced by 50 nM CXCL12, both after pre-incubation with the indicated test compound. Values are calculated relative to the reference response i.e. CXCL12 response without compound pre-incubation. Compounds were pre-incubated for 1 h except for PTX and YM-254890 which were pre-incubated overnight. The mean and standard deviation from 3 independent experiments are shown. Within each experiment at least two technical replicates for each condition are averaged. The different compounds are listed with their targets in the text box underneath the figure. P-values were determined using one sample t-tests with null hypothesis stating that the response values after compound pre-incubation did not differ from the reference response value 100. A correction for multiple testing was performed based on the Benjamini-Hochberg procedure. * indicates significant differences between responses with compound pre-incubation and the reference response value 100 for FDR = 0.1. Calcium fluxes were measured using the FLIPR tetra system with the calcium-sensitive dye Fluo-2 AM whereas the impedance measurements were done using the xCELLigence device.

In order to unravel the contribution of G-protein signaling and other downstream signaling cascades previously described for CXCR4 [31–33], the effect of small molecule inhibitors on the CXCR4-specific cellular electric impedance response was investigated. To avoid unspecific, CXCR4-unrelated effects of the applied compounds, only concentrations of compound that did not evoke strong deviations in the impedance during the pre-incubation period (*i*.*e*. in the absence of CXCL12 stimulation) were used (see statistical analysis section and **S5 Fig**). Because most compounds were dissolved in DMSO, the potential effect of the final DMSO concentration was also taken into account in the analysis. To determine whether a compound had a significant effect on the CXCR4 impedance response, one sample t-tests were performed with null hypothesis stating that the response values following compound pre-incubation did not differ from the reference response value. A correction for multiple testing was done using the Benjamini-Hochberg procedure with FDR = 0.1. Pertusis toxin (PTX), known to specifically prevent G_αi_-signaling, or YM-254890, which specifically inhibits G_αq_ [34], was added after a cell monolayer had formed and neither disturbed the cells during overnight pre-incubation. When pre-incubated overnight with PTX (50 ng/mL, final concentration), the CXCL12-induced impedance response was completely abolished, while pre-incubation with YM-254890 had only a moderate, and not significant, effect on the CXCR4 response (**Fig 3**). Pre-incubation with PTX and YM-254890 was also investigated in a cell-based assay measuring CXCL12-induced calcium mobilization. Here, both PTX (overnight pre-incubation) and YM-254890 (overnight and one-hour pre-incubation) had significant effects. They strongly inhibited the CXCL12-induced (50 nM, final concentration) calcium release, suggesting that both G_αi_ and G_αq_ are involved in the CXCR4-mediated calcium signaling (**Fig 3**). However, when measuring electric impedance responses, G_αi_ appeared to be the dominant G-protein as G_αq_ inhibition had only a minor effect. The contribution of G_βγ_-dependent signaling to the CXCR4 cellular impedance response was determined by pre-incubating cells for one hour with the G_βγ_-inhibitor gallein (10 μM, final concentration) [35]. This led to a small but significant reduction in the impedance response. No clear effect of gallein was observed on the calcium response. Overnight pre-incubation with gallein did not diminish the impedance or calcium response to a further extent. Several other compounds, known to inhibit target molecules involved in more “distal” signaling steps (e.g. MEK1/2 and PI3K), were also tested. These compounds did not significantly modify the CXCR4-specific electric impedance response (**Fig 3**).

### CXCR7 expression strongly impairs the CXCR4-mediated cellular electric impedance response

**Fig 4:**
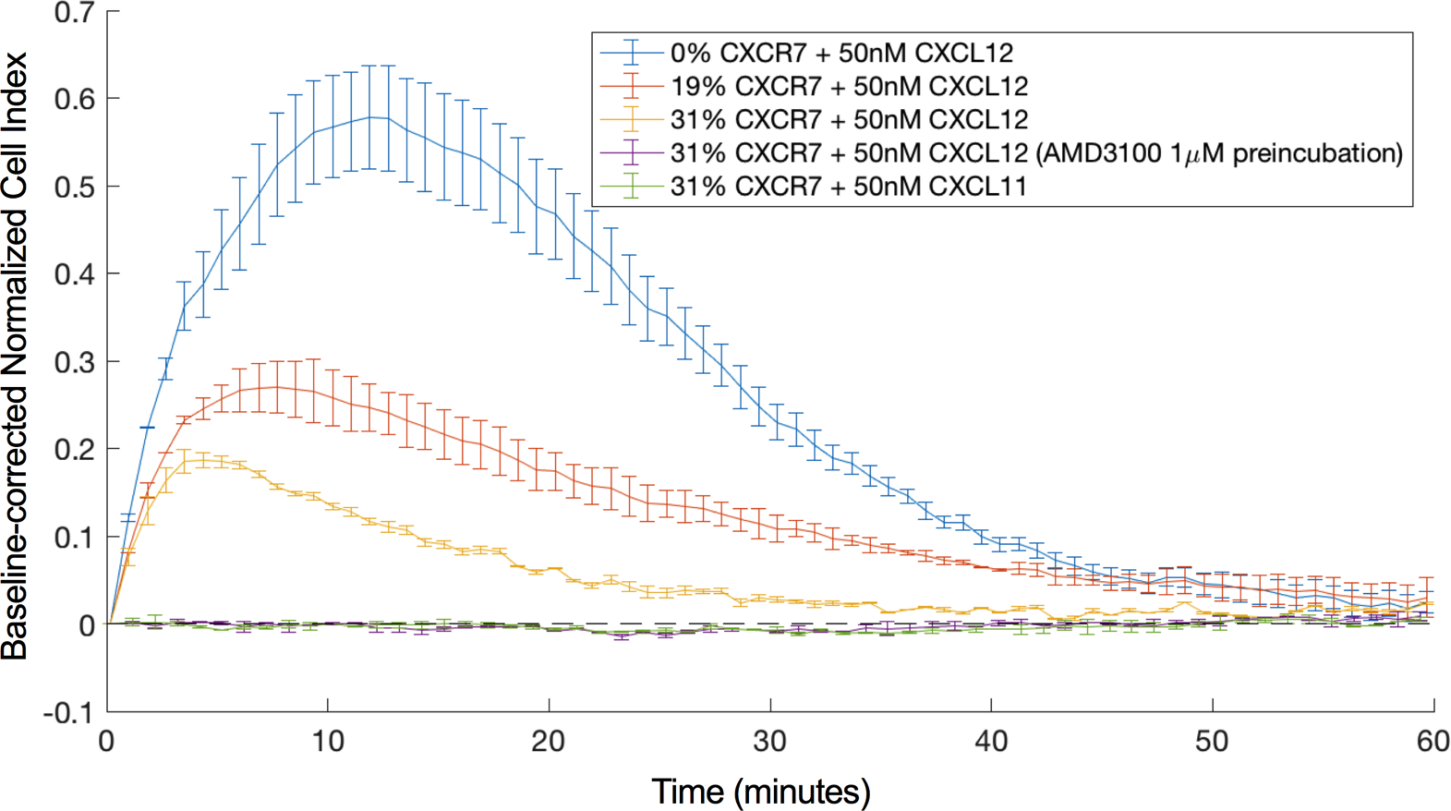
Impedance response following stimulation with 50 nM CXCL12 of U87.CD4.CXCR4 cells transfected with varying amounts of CXCR7. The effect of 0% (blue), 19% (orange) and 31% (yellow) CXCR7 co-expressing cells on the CXCL12 induced CXCR4 response is shown. The response after stimulation with 50 nM CXCL11 (I-TAC) (green) and the CXCL12-induced response after pre-incubation with 1 μM AMD3100 (purple) are shown as well. Mean and standard deviation were estimated from two technical repeats. This figure represents one of the datasets used for the linear least square regression that is depicted in Fig 5. For clarity, one in every 5 data points is shown.

**Fig 5:**
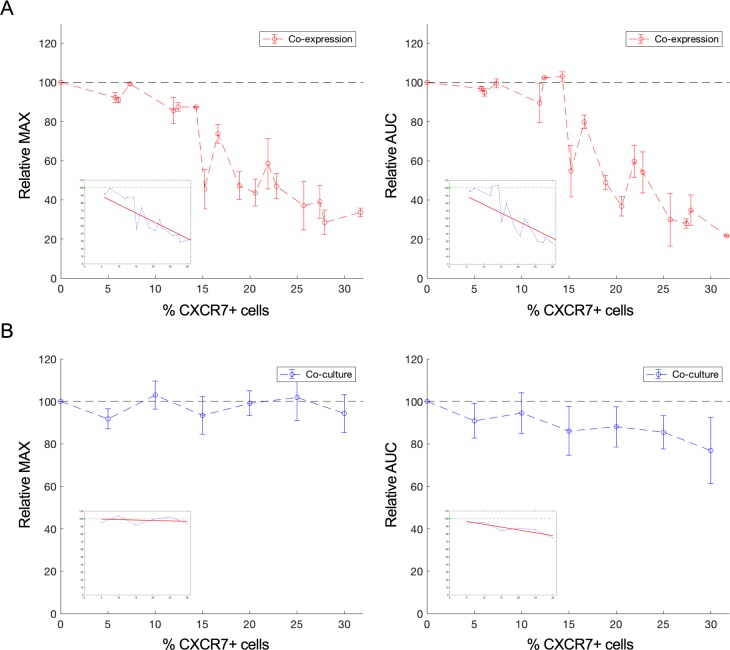
Relative MAX and AUC of the CXCL12 induced CXCR4 response in function of CXCR7 expressing cells. **(a)** Co-expression cultures, in which CXCR4 and CXCR7 are co-expressed on the cell surface. Relative MAX response (left) and relative AUC (right) within the first 60 minutes after ligand addition (50 nM CXCL12) relative to the level of CXCR7 co-expression in U87.CD4.CXCR4 cells (red). The response of U87.CD4.CXCR4 cells solely transfected with empty vector plasmid (CXCR7 not present) was set to 100%. Data from six independent experiments (with slightly varying transfection efficiencies) are included in the analysis. Mean and standard deviation were estimated from two technical repeats within each experiment **(b**) Co-cultures in which CXCR4 and CXCR7 were not co-expressed on the same cell but present on different cell populations. Relative MAX response (left) and relative AUC (right) within the first 60 minutes after ligand addition (50 nM CXCL12) to U87 co-cultures with increasing amount of X4^-^/X7^+^ cells (blue). For each ratio of X4^+^/X7^-^ in co-culture with X4^-^/X7^+^ cells, the response of a similar ratio of X4^+^/X7^-^ cells in co-culture with X4^-^/X7^-^ cells was set to 100%. The mean and standard deviation were calculated using three independent experiments. Within each experiment, two technical repeats were averaged. Linear least square regression analysis was performed on the relative AUC and MAX values in function of the % CXCR7-positive cells and the best linear fit for the data is shown in each figure.

CXCR4 and CXCR7 are able to form heterodimers whereby CXCR7 modulates the signaling properties of CXCR4, for instance its ability to mobilize cytosolic calcium [18]. Here, we investigated the effect of increasing CXCR7 expression levels on the CXCR4-mediated cellular electric impedance response. For this, U87.CD4.CXCR4 cells were transiently transfected with increasing amounts of a CXCR7-encoding plasmid. CXCR7 receptor expression levels were quantified by flow cytometry three days post transfection (**S4 Fig**). Two days post transfection, cells were seeded in an E-Plate VIEW 16 PET and grown overnight. The next day, cells were stimulated with 50 nM CXCL12 as described before. CXCR7 expression was able to decrease the CXCL12-induced impedance response (**Figs 4 and 5A**) while leaving the cell surface level of CXCR4 unchanged (**S4 Fig**).

When looking at the MAX as a function of the CXCR7 expression level, the best linear fit for the data is given by the equation *y* = −2.219 + 100. For the AUC as a function of the CXCR7 expression level, the best fit is given by *y* = −2.195 + 100. When performing F-tests on these data with the null hypothesis stating that y = 100 (increasing amounts of CXCR7 do not diminish the response), F-values of 227.1 and 109.9 were obtained for MAX and AUC, respectively, indicating a significant negative correlation between the CXCR4-mediated response and the percentage of CXCR7-positive cells at significance level 0.05 with p-values lower than 0.01. This reduced response could still be fully inhibited by pre-incubation with AMD3100, which indicates that the response results from CXCR4 stimulation. Also, stimulation with 50 nM CXCL11 of U87.CD4.CXCR4 cells co-expressing CXCR7 did not evoke a detectable response, indicating that co-expression did not enhance a detectable CXCR7-mediated response (**Fig 4**). Thus, although the presence of CXCR7 clearly modulated the CXCR4 response, the presence of CXCR4 in turn did not give rise to a detectable CXCR7 impedance response.

### Co-culturing U87.CD4.CXCR4 and U87.CD4.CXCR7 cells has only a limited impact on the CXCR4-mediated response

Given its high affinity for CXCL12, CXCR7 is believed to control the bioavailability of this chemokine by scavenging [5,6,36]. To study the possible effect of scavenging by CXCR7 on the impedance response of CXCR4, we used co-cultures of U87.CD4.CXCR4 (X4^+^/X7^-^) and U87.CD4.CXCR7 (X4^-^/X7^+^) cells. It is assumed that CXCR4 and CXCR7 cannot form heterodimers as they are separately expressed on different cell populations. The total number of cells per well was similar to the transfected cultures, but the number of CXCR4-positive cells was less due to the presence of varying amounts of U87.CD4 (X4^-^/X7^-^) or U87.CD4.CXCR7 (X4^-^/X7^+^) cells. Co-cultures of X4^+^/X7^-^ with X4^-^/X7^+^ were always compared to co-cultures consisting of equal amounts of X4^+^/X7^-^, but with X4^-^/X7^-^ cells. The response MAX and AUC of the X4^+^/X7^-^ with X4^-^/X7^-^ co-culture (thus without CXCR7-positive cells) was seen as the reference value and set to 100% for each population ratio. The response MAX and AUC of the co-culture with a similar X4^+^/X7^-^ population size but co-cultured with X4^-^/X7^+^ cells instead of the X4^-^/X7^-^ cells was set relative to the reference value. This was done to take into account the decrease in CXCR4-positive cells when increasing the CXCR7-positive cell population. The growth curves of the different co-cultures were comparable, which suggests that the subpopulations grew at similar rates. Using flow cytometry, it was further confirmed that the X4^+^/X7^-^ with X4^-^/X7^-^ and X4^+^/X7^-^ with X4^-^/X7^+^ co-cultures remained stable overnight and that no receptor exchange occurs as no cells were found to be positive for both CXCR4 and CXCR7.

In **Fig 5B**, the relative MAX and the AUC for different ratios of CXCR4-positive to CXCR7-positive cells is presented. The best fit through the relative MAX as a function of the fraction of CXCR7-positive cells is given by the equation *y* = −0.114 + 100 whereas for the relative AUC as a function of the fraction of CXCR7-positive cells this is given by *y* = −0.743 + 100. F-tests were performed with null hypothesis stating that y = 100. For MAX (F-value = 1.1), the negative trend was not significant at significance level 0.05 (p = 0.44), whereas for AUC (F-value = 97.9), a significant negative correlation was found between the percentage of U87.CD4.CXCR7 cells and the AUC within the first 60 minutes after ligand addition (p < 0.01). The correlation between the relative AUC values and % CXCR7 expression suggests that there is a small response-reducing effect of scavenging. However, since the negative trend and correlation are much more pronounced in the co-expression experiment, it is suggested that besides scavenging, additional mechanisms underlie the diminished CXCR4-mediated response in the presence of CXCR7.

## Discussion

Pharmacological and functional cell-based assays are important tools in drug discovery as they can be used to identify and profile small molecules or biologics that modulate the activity of disease-relevant chemokine receptors such as CXCR4 and CXCR7. Cellular electric impedance spectroscopy is a label-free, non-invasive technique allowing the recording of an “integrative” biological response which, in contrast to many existing assays, does not depend on the activation of a single particular signaling pathway or on a single readout. In this report, cellular electric impedance spectroscopy was evaluated as an alternative method for measuring agonist-induced CXCR4 and CXCR7 activity. Whereas for CXCR4 dose-dependent and specific impedance responses could be detected, no activity was recorded when CXCR7 was stimulated with its natural agonists, the chemokines CXCL11 and CXCL12. Previous reports have demonstrated the lack of G-protein signaling via CXCR7 [6], but CXCR7 is able to recruit β-arrestin under basal conditions and after agonist stimulation [37]. The fact that no CXCR7-mediated electric impedance response was detected supports the idea that G-protein signaling is the major contributor to the impedance readout while β-arrestin recruitment would have little to no effect. This is further supported by the finding that the CXCR4 impedance response can be completely blocked by the G_αi_ protein inhibitor PTX. Previous research on the dopamine receptor 1 indicated that ligands, failing to internalize the receptor, had a similar impedance profile as ligands that extensively induced receptor internalization. Since internalization often requires β-arrestin recruitment, this study supports the possibility that G-protein independent signaling is not detected using impedance measurements [38]. In contrast, Kammermann et al. (2011) reported that for GPR109A the observed impedance minimum was mediated by the β-arrestin pathway [39].

CXCR7 is believed to control the *in vivo* bioavailability of CXCL12, for instance, to avoid overstimulation and concomitant internalization of CXCR4 [40]. Also, heterodimerization and crosstalk between CXCR4 and CXCR7 have been described previously [17,41], but with some conflicting results. Whereas in some studies, CXCR7 decreased the CXCR4-induced calcium mobilization [17], other studies have reported a stimulatory effect of CXCR7 on CXCR4-mediated calcium release [18]. Using cellular electric impedance spectroscopy, a significant negative correlation between the CXCR4-mediated impedance response and the level of co-expressed CXCR7 was observed. Understanding the mechanism behind the negative impact of CXCR7 on CXCR4 signaling requires further investigation, as it can be diverse. Scavenging of CXCL12 by CXCR7 might contribute to the observed effect. However, in the described co-culture experiments only a very mild effect of CXCR7 on the CXCR4-mediated response was observed compared to the co-expression experiments. Therefore, our data suggests that additional mechanisms are involved. These might include the stabilization of a less active CXCR4 conformation resulting in a shift in G-protein-coupling, induced upon dimerization with CXCR7 [15] or an increased level of internalization of the CXCR4-CXCR7 dimer compared to CXCR4 alone. It could also be that the availability of G_αi_ for CXCR4 alters since CXCR7 might interact with G_αi_ as well [17]. The diminished response could still be blocked with AMD3100, and no response was observed when stimulating the co-transfected cells with CXCL11, suggesting that the response is CXCR4-specific.

The colorectal cancer cell line HT-29 endogenously expresses CXCR4 (~36% of cells are CXCR4-positive) while practically no CXCR7 is expressed in these cells. Fifty nM CXCL12 evoked a small, but clearly detectable impedance response on these cells, which reaches its maximum up to 10 times faster than in U87.CD4.CXCR4 cells. After enriching the CXCR4-positive population, a ~90% CXCR4-positive HT-29 population was obtained, that showed a more intense CXCL12-induced impedance response, which again reached its maximum much faster than a similar response on U87.CD4.CXCR4 cells. Of interest, the response intensity was still much lower than the corresponding response on U87.CD4.CXCR4 cells. Of note, flow cytometry data demonstrated that HT-29 cells are less intensely stained compared to U87.CD4.CXCR4 cells indicating that less receptor per cell is present, which might explain the lower readout in cellular electric impedance (**Fig 2C**). In addition, different availabilities of downstream effectors can be responsible for the observed effect. Aside from the shift in response kinetics, both cell types have a similar CXCL12-induced CXCR4 response profile. Princen et al. (2003) previously showed that the CXCL12-induced calcium response magnitude is also related to the CXCR4 expression level [31]. CXCL12 stimulation of the original and the CXCR4-enriched HT-29 cell type did not evoke an eminent calcium response on the FLIPR Tetra. This suggests that cellular electric impedance spectroscopy is more sensitive for detecting endogenous CXCR4 signaling than the FLIPR Tetra.

The identification of biased ligands (including biased antagonists), that preferentially block one particular signaling or functional pathway over the other, generally requires the evaluation of compounds in multiple receptor-related functional or pharmacological assays. By dissecting the integrated cellular electric impedance response upon CXCR4 activation with pathway-specific small molecule inhibitors, we wanted to evaluate if cellular electric impedance readouts could be useful for identifying such biased ligands. It has been reported that both Extracellular Signal-Regulated Kinases 1/2 (ERK 1/2) and Akt are involved in CXCL12-induced proliferation of glioblastoma cells [42]. However, compounds that inhibited the MEK1/2 and PI3K-Akt pathways downstream of CXCR4 (PD98059, U0126 & LY294002) had no or little effect on the impedance response, at least at the concentrations that were tested. On the other hand, some compounds acting more “upstream” on the signaling cascade (e.g. at the G-protein and receptor level) had pronounced effects on the impedance response elicited by CXCL12, as well cytochalasin B, which affects the cell’s morphology within the first hour after addition [43]. Using these compounds, we showed that G_αi_-induced processes strongly influence the impedance response, whereas G_αq_-induced processes have no to only minor effects on the impedance profile of CXCR4. Since both of these compounds strongly decreased the CXCL12 induced calcium response, it seems that calcium signaling has little influence on the impedance response of CXCR4. G_βγ_-induced events contribute to the impedance response as well, but the effect is limited. Higher concentrations of gallein can decrease the response further, but the relevance of these higher concentrations is questionable due to their strong effect on the electric impedance during the pre-incubation phase. Further research must clarify whether the contribution of the different pathways to the impedance response can be generalized to other GPCRs as well.

Even though cellular electric impedance spectroscopy can, to a certain extent, be used to distinguish and explore different agonists [44,45], subtle effects of biased ligands on CXCR4’s downstream signaling pathways might not be readily detectable. Perhaps this is due to increasing bifurcation and divergence in pathways, so that blocking a single downstream branch in a complex network cannot block the entire cellular response anymore.

The CXCR4 response profile is similar to the previously reported CXCR3 profile [45], which is another chemokine receptor that is coupled to G_αi_. Whether the profile’s shape can be generalized to other chemokine G_αi_-coupled receptors as well, and whether similar intracellular events contribute to the profile will require further research.

## Conclusions

Cellular electric impedance spectroscopy proves to be a valuable pharmacological assay with added value compared to many existing GPCR assays. It can be used to explore GPCR-related events as its integrated readout can capture different aspects of the overall cellular response. Being able to evaluate the compound pre-incubation phase is an interesting feature for drug development as it can help determine immediate off-target effects of compounds, and can give clues about drug toxicity over longer periods of time. Moreover, it is a valuable receptor assay, as it can detect endogenous receptor activation where other assays might fail. The impedance responses are also suitable for more in depth bio-informatics analysis *e*.*g*. one could explore response kinetics using a variety of modeling techniques.

More research will be required to explore which mechanisms besides scavenging diminish the CXCR4 impedance response to CXCL12 in the presence of CXCR7. As mentioned before, CXCR4 and CXCR7 are involved in various forms of cancer and their metastasis. Hence, a better understanding of these two GPCRs and how they interact may assist in the development of new cancer treatments.

## Supporting information

S1 Fig Electric impedance profile of growth and attachment of U87.CD4.CXCR4 cells. An average of 16 technical repeats is shown as a black line and the grey zone indicates the standard deviation. The CI was measured every 20 minutes.
(TIFF)

S2 Fig Electric impedance profile of U87.CD4.CXCR7 cells stimulated by CXCL12 (orange) or CXCL11 (yellow). The responses are baseline-corrected to a medium response without ligand (blue). Mean and standard deviation are shown over 6 technical repeats from one experiment. One in every 5 data points is shown for clarity.
(TIFF)

S3 Fig CXCR4 expression level of original (blue) and enriched (red) HT-29 cells determined by flow cytometry. The cells were stained using APC mouse anti-human CXCR4. The APC-A+ gate was chosen using a control APC antibody not specific for CXCR4 (grey). The percentage of CXCR4-positive cells was 36% for the original HT-29 population and 89.9% for the enriched population.
(TIFF)

S4 Fig CXCR4 and CXCR7 expression levels in transfected U87.CD4.CXCR4 cells determined by flow cytometry. (**a**) Dot plots based on PE (CXCR7) and APC (CXCR4) signal of the cells. From left to right a higher quantity of pBABE/puro CXCR7 plasmid was transfected (cyan—orange—green). In red, the distribution of cells transfected solely with pBABE/puro EV is shown. In grey the isotype staining of empty-vector-transfected U87.CD4.CXCR4 cells is shown. Q1 and Q3 represent CXCR7- and CXCR4-positive populations, respectively. The Q2 population represents cells that are both CXCR4- and CXCR7-positive. The four groups (Q1-Q4) are separated using the spread of the isotype population (grey) and the CXCR4 and CXCR7 co-stained empty-vector-transfected U87.CD4.CXCR4 cells (red). **(b)** Left: Histogram showing that CXCR4 surface expression remains more or less constant after transfection with varying amounts of CXCR7 (mean APC values are shown in the table). Middle: histogram showing that the PE-positive population increases when more CXCR7 plasmid is transfected (mean PE-values are shown in the table). Right: histogram showing the double-positive fractions of the different transfections. The amount of CXCR4 was determined using APC-labeled mouse anti-human CD184, while the amount of CXCR7 was determined using PE-labeled mouse anti human CXCR7.
(TIFF)

S5 Fig CXCL12-elicited impedance response on U87.CD4.CXCR4 cells [0–120 min] including compound pre-incubation period [-60-0 min]. The response to 10 μM LY294002 (orange, dashed) lies outside the trust interval, and this concentration of LY294002 is therefore not included in the analysis. The CXCL12 response without LY294002 pre-incubation (blue, full) and with 2μM LY294002 pre-incubation (pink, full) lie inside the interval and are included in the analysis. Responses are normalized on the point of ligand addition. The trust interval was created using the mean +/- 3 standard deviations of 16 control responses (no compound pre-incubation) of four independent experiments. The trust interval is depicted in the figure as a grey zone in the pre-incubation period. Compound concentrations that fall outside of this interval have a strong effect on the electric impedance by themselves, and are not included in the analysis.
(TIFF)

S6 Fig Calcium responses on enriched HT-29 cells (90% CXCR4-positive) elicited by CXCR4 and PAR1 activation. The response to 20 μM digitonin was set to 100% (maximal calcium release) and the other ligand responses relative to this value. HT-29 is known to signal via PAR1 [1]. Even though, clear calcium fluxes could be detected after PAR1 stimulation using thrombin and PAR1-AP, no eminent response is detected when 50nM CXCL12 is applied. The figure represents four technical replicates. P-values were determined based on one sample t-tests with null hypothesis stating that the response values did not differ from the baseline response with value 0. A correction for multiple testing was performed using the Benjamini-Hochberg procedure. * indicates significant differences from the baseline response for FDR = 0.1. Calcium fluxes were measured using the FLIPR tetra system with the calcium sensitive dye fluo-2 AM.
[1] Darmoul D, Gratio V, Devaud H, Lehy T, Laburthe M. Aberrant expression and activation of the thrombin receptor protease-activated receptor-1 induces cell proliferation and motility in human colon cancer cells. Am J Pathol. 2003 May;162(5):1503–13.
(TIFF)

## Abbreviations

ACKR3: Atypical Chemokine Receptor 3
AUC: Area Under the Curve
CI: Cell Index
CXCL11: CXC Chemokine Ligand 11
CXCL12: CXC Chemokine Ligand 12
HIV: Human Immunodeficiency Virus
I-TAC: Interferon-inducible T-cell Alpha Chemokine
MAX: Maximum response intensity
MEK: Mitogen-activated protein kinase kinase
PTX: Pertussis Toxin
PI3K: Phosphatidylinositol-3-Kinase
WHIM: Warts Hypogammaglobulinemia, Immunodeficiency, and Myelokathexis
