# Supplementary figures and images for: Signaling properties of the human chemokine receptors CXCR4 and CXCR7 by cellular electric impedance measurements
Source: PLoS One. 2017 Sep 25;12(9):e0185354. doi: 10.1371/journal.pone.0185354 (PMC5612718; doi:10.1371/journal.pone.0185354)

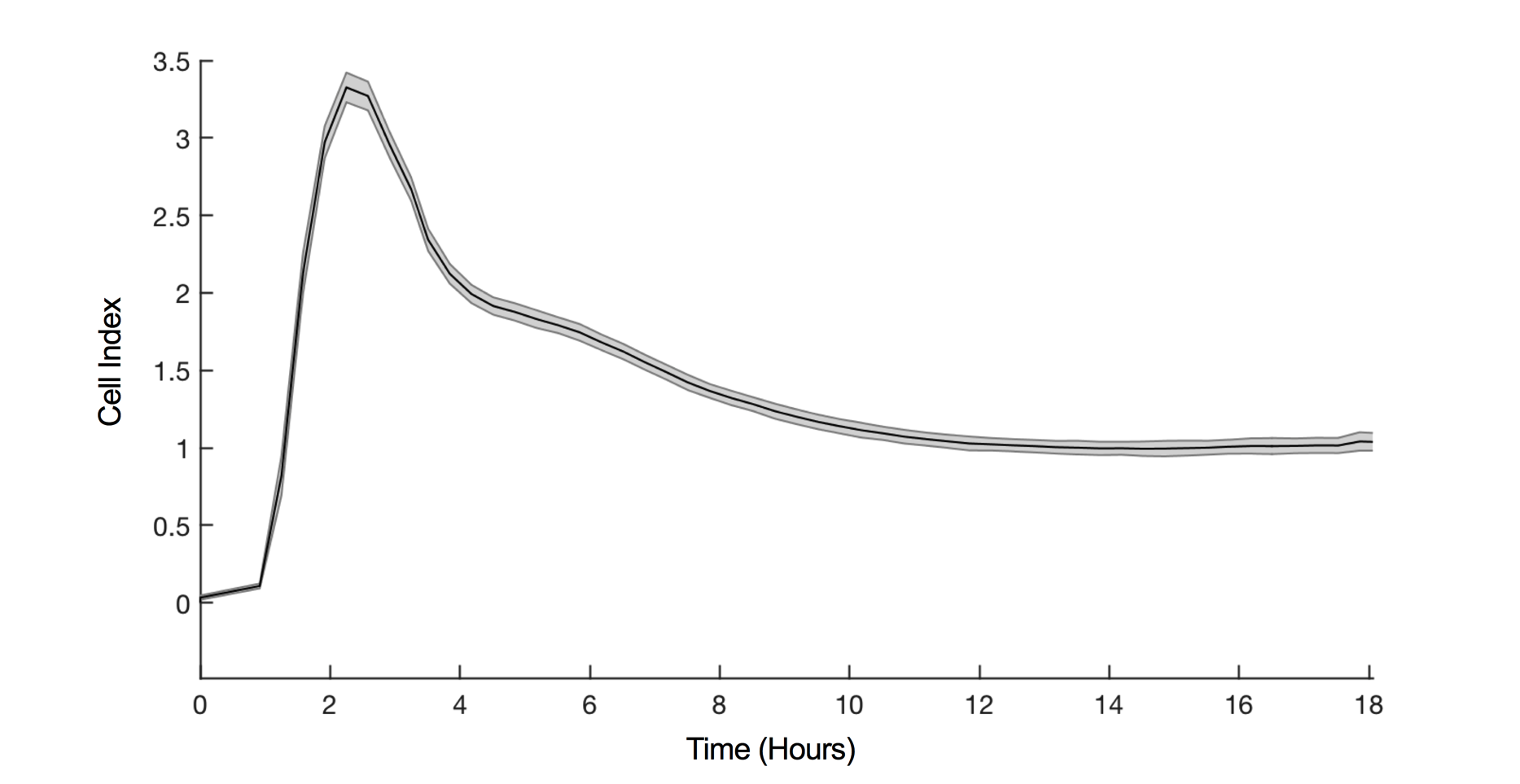

Supplement: S1 Fig — An average of 16 technical repeats is shown as a black line and the grey zone indicates the standard deviation. The CI was measured every 20 minutes. (TIFF) [file pone.0185354.s001.tiff]

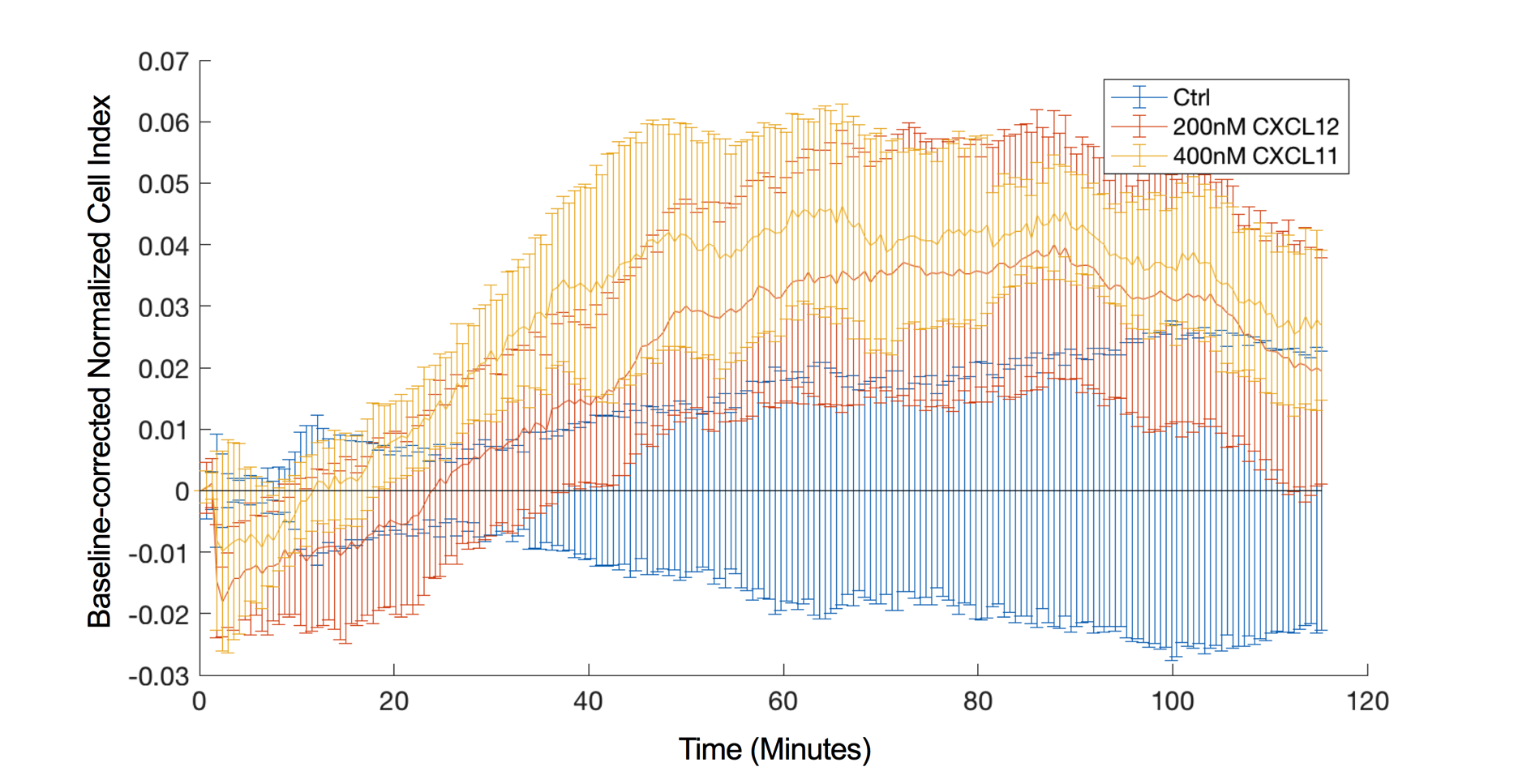

Supplement: S2 Fig — The responses are baseline-corrected to a medium response without ligand (blue). Mean and standard deviation are shown over 6 technical repeats from one experiment. One in every 5 data points is shown for clarity. (TIFF) [file pone.0185354.s002.tiff]

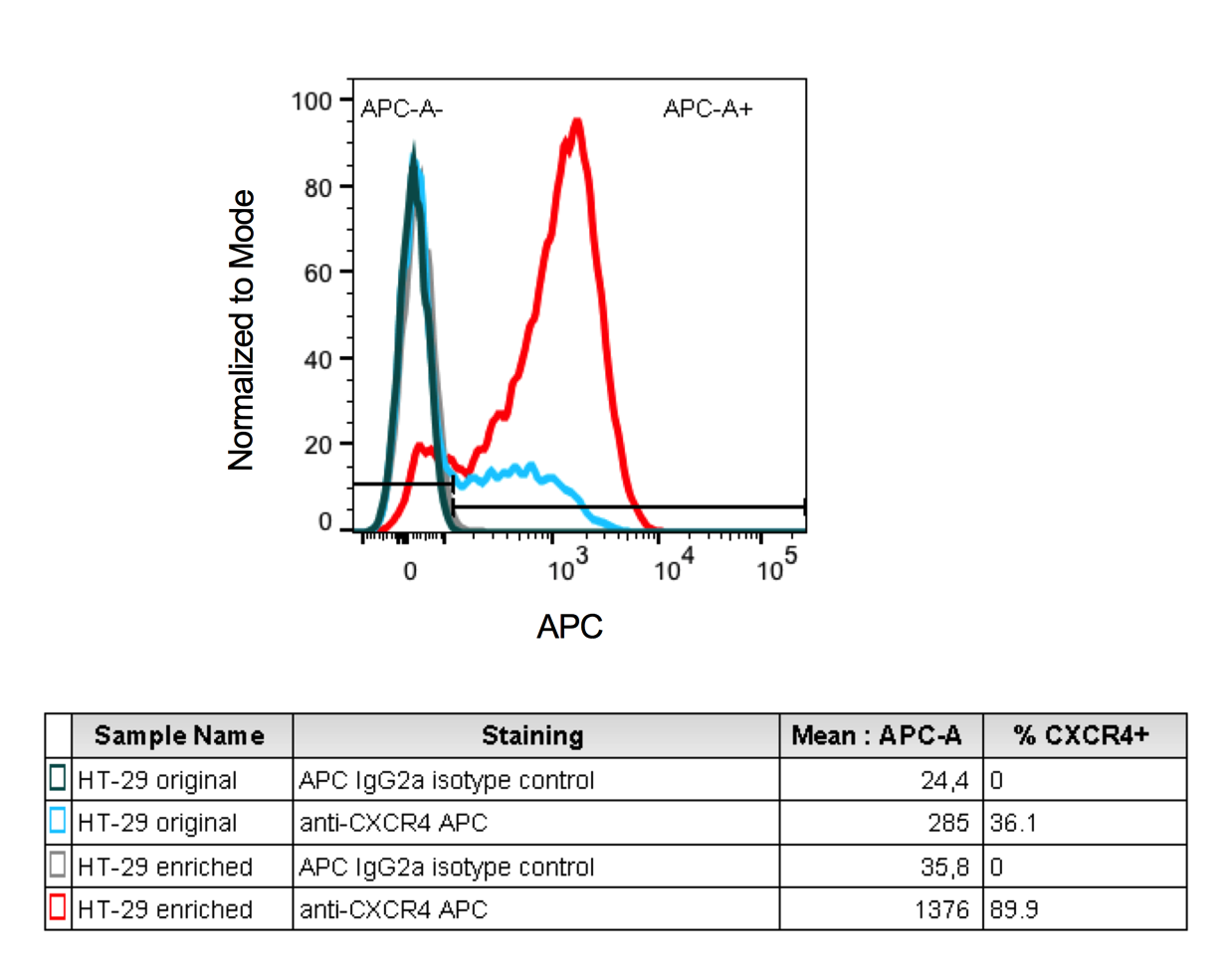

Supplement: S3 Fig — The cells were stained using APC mouse anti-human CXCR4. The APC-A+ gate was chosen using a control APC antibody not specific for CXCR4 (grey). The percentage of CXCR4-positive cells was 36% for the original HT-29 population and 89.9% for the enriched population. (TIFF) [file pone.0185354.s003.tiff]

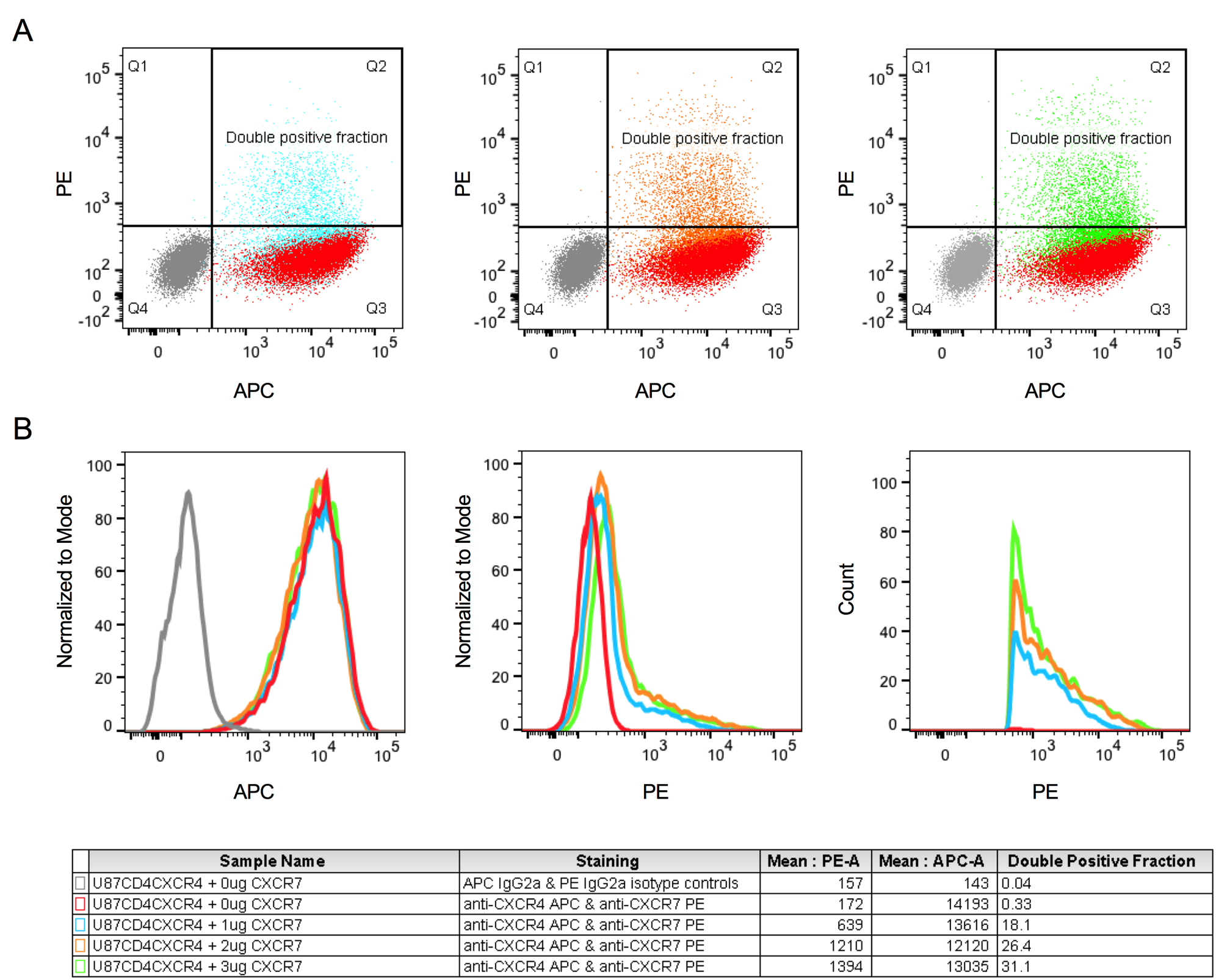

Supplement: S4 Fig — (a) Dot plots based on PE (CXCR7) and APC (CXCR4) signal of the cells. From left to right a higher quantity of pBABE/puro CXCR7 plasmid was transfected (cyan—orange—green). In red, the distribution of cells transfected solely with pBABE/puro EV is shown. In grey the isotype staining of empty-vector-transfected U87.CD4.CXCR4 cells is shown. Q1 and Q3 represent CXCR7- and CXCR4-positive populations, respectively. The Q2 population represents cells that are both CXCR4- and CXCR7-positive. The four groups (Q1-Q4) are separated using the spread of the isotype population (grey) and the CXCR4 and CXCR7 co-stained empty-vector-transfected U87.CD4.CXCR4 cells (red). (b) Left: Histogram showing that CXCR4 surface expression remains more or less constant after transfection with varying amounts of CXCR7 (mean APC values are shown in the table). Middle: histogram showing that the PE-positive population increases when more CXCR7 plasmid is transfected (mean PE-values are shown in the table). Right: histogram showing the double-positive fractions of the different transfections. The amount of CXCR4 was determined using APC-labeled mouse anti-human CD184, while the amount of CXCR7 was determined using PE-labeled mouse anti human CXCR7. (TIFF) [file pone.0185354.s004.tiff]

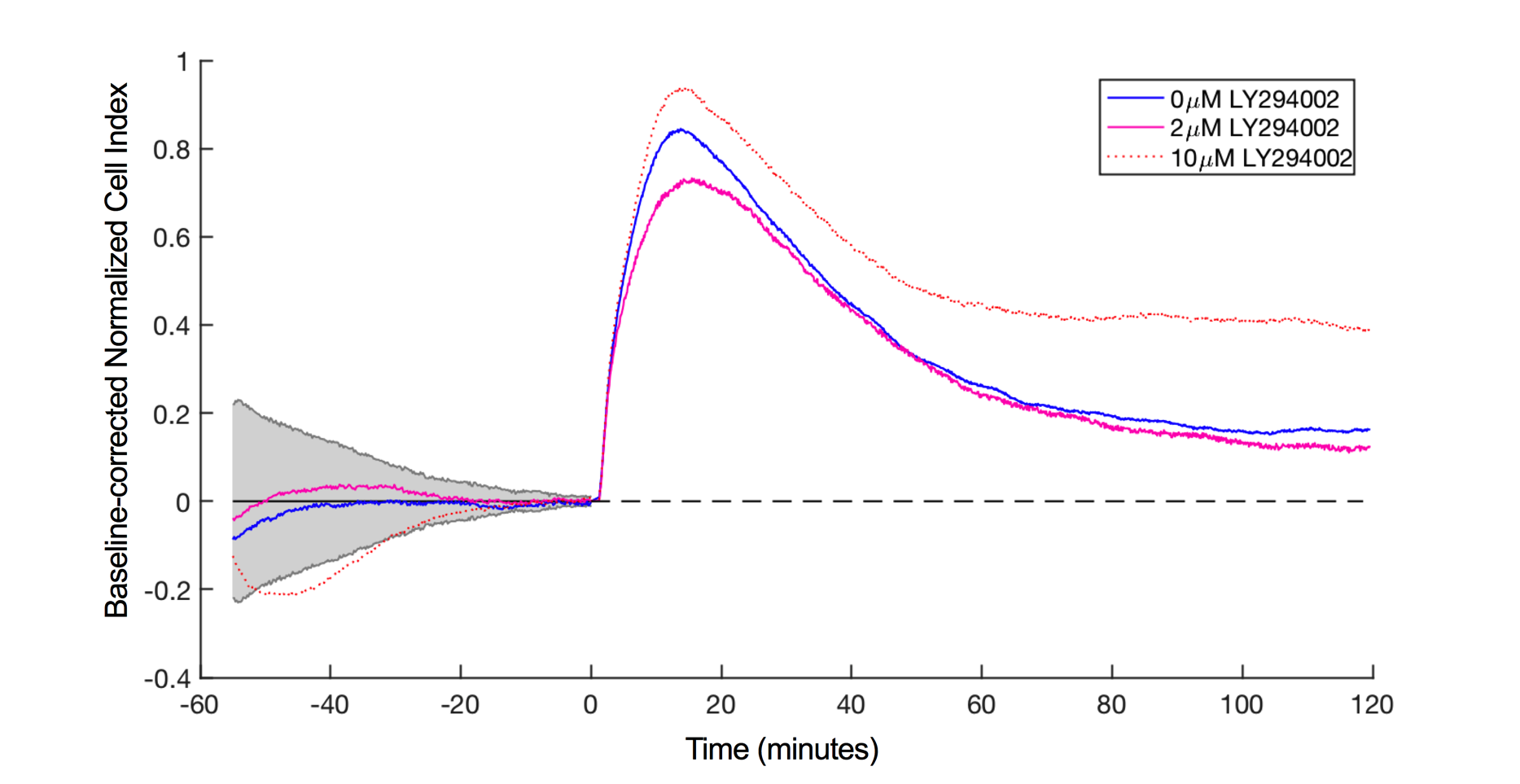

Supplement: S5 Fig — The response to 10 μM LY294002 (orange, dashed) lies outside the trust interval, and this concentration of LY294002 is therefore not included in the analysis. The CXCL12 response without LY294002 pre-incubation (blue, full) and with 2μM LY294002 pre-incubation (pink, full) lie inside the interval and are included in the analysis. Responses are normalized on the point of ligand addition. The trust interval was created using the mean +/- 3 standard deviations of 16 control responses (no compound pre-incubation) of four independent experiments. The trust interval is depicted in the figure as a grey zone in the pre-incubation period. Compound concentrations that fall outside of this interval have a strong effect on the electric impedance by themselves, and are not included in the analysis. (TIFF) [file pone.0185354.s005.tiff]

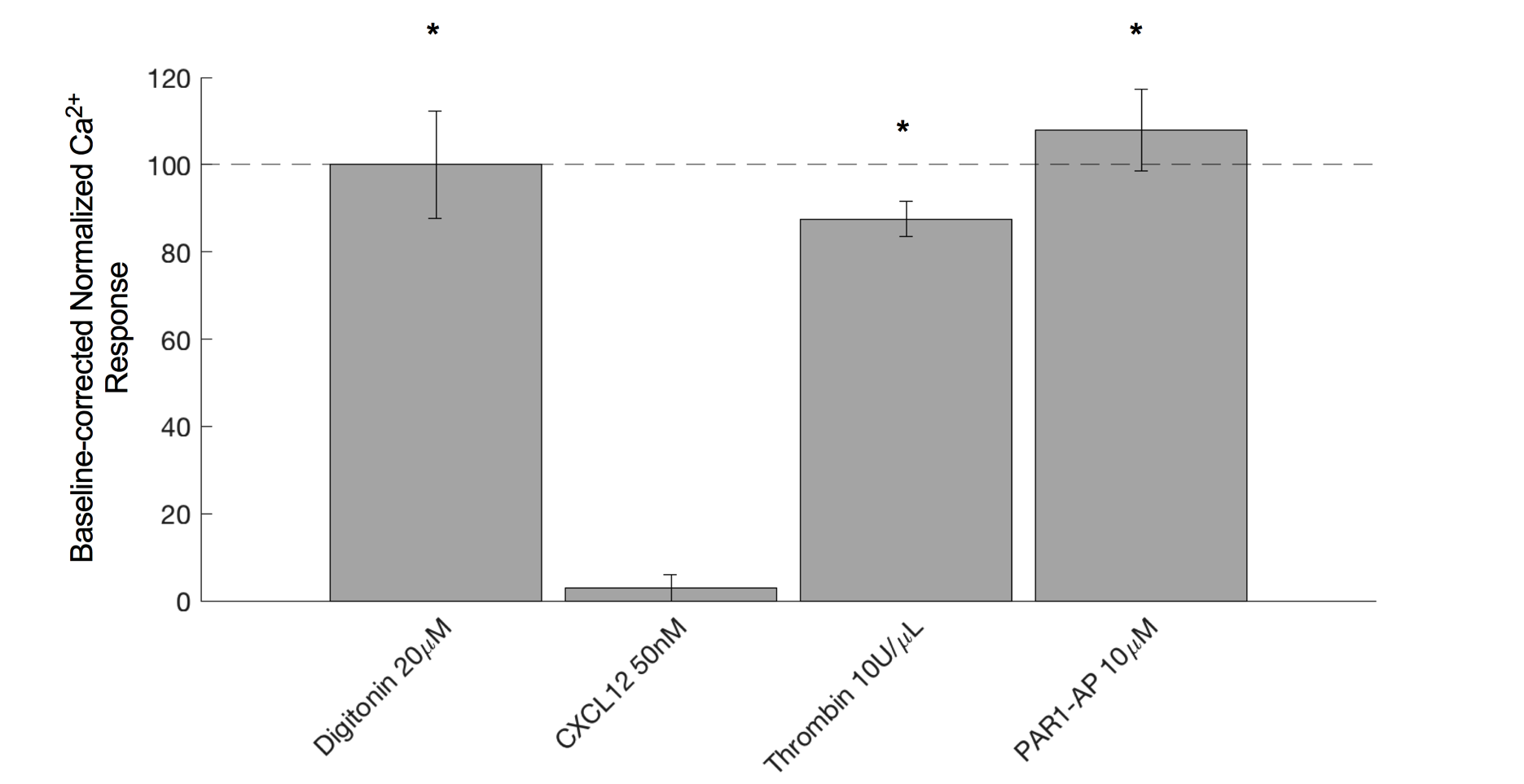

Supplement: S6 Fig — The response to 20 μM digitonin was set to 100% (maximal calcium release) and the other ligand responses relative to this value. HT-29 is known to signal via PAR1 [1]. Even though, clear calcium fluxes could be detected after PAR1 stimulation using thrombin and PAR1-AP, no eminent response is detected when 50nM CXCL12 is applied. The figure represents four technical replicates. P-values were determined based on one sample t-tests with null hypothesis stating that the response values did not differ from the baseline response with value 0. A correction for multiple testing was performed using the Benjamini-Hochberg procedure. * indicates significant differences from the baseline response for FDR = 0.1. Calcium fluxes were measured using the FLIPR tetra system with the calcium sensitive dye fluo-2 AM. [1] Darmoul D, Gratio V, Devaud H, Lehy T, Laburthe M. Aberrant expression and activation of the thrombin receptor protease-activated receptor-1 induces cell proliferation and motility in human colon cancer cells. Am J Pathol. 2003 May;162(5):1503–13. (TIFF) [file pone.0185354.s006.tiff]
